# Triple-negative breast cancer: from none to multiple therapeutic targets in two decades

**DOI:** 10.3389/fonc.2023.1244781

**Published:** 2023-11-09

**Authors:** Filomena Marino Carvalho

**Affiliations:** Department of Pathology, Faculdade de Medicina FMUSP, Universidade de Sao Paulo, Sao Paulo, SP, Brazil

**Keywords:** breast cancer, TNBC, biomarkers, immunotherapy, PARP inhibitors, ADCS, HER2-low, TROP2

## Abstract

Triple-negative breast cancers (TNBCs) are more likely to occur in younger patients and have a poor prognosis. They are highly heterogeneous tumors consisting of different molecular subtypes. The only common characteristic among them is the absence of targets for endocrine therapy and human epidermal growth factor receptor 2 (HER2) blockade. In the past two decades, there has been an increased understanding of these tumors from a molecular perspective, leading to their stratification according to new therapeutic strategies. TNBC has ushered breast carcinomas into the era of immunotherapy. The higher frequency of germline BRCA mutations in these tumors enables targeting this repair defect by drugs like PARP inhibitors, resulting in synthetic lethality in neoplastic cells. Additionally, we have the identification of new molecules to which this generation of smart drugs, such as antibody-drug conjugates (ADCs), are directed. In this review, we will discuss the trajectory of this knowledge in a systematic manner, presenting the molecular bases, therapeutic possibilities, and biomarkers.

## 1 Introduction

Breast cancer accounts for 31% of female cancers in the United States, with 297,540 estimated new cases in 2023 and 43,170 deaths (1). Triple-negative breast cancers (TNBCs) correspond to about 15% of the cases. They are characterized by a lack of targets for endocrine therapy and human epidermal growth factor receptor 2 (HER2) blockage, specifically with <1% expression of hormone receptors (HRs) and HER2-negative (2). The group is very heterogeneous, as one can expect from tumors defined by negative features. Most TNBCs are high-grade invasive carcinomas of no special type (NST). However, rare special histologies, such as salivary gland-type tumors, metaplastic carcinoma, and others, even less frequent, can be found (2). Low-risk tumors, with no/minimal metastatic potential, are rare among TNBC. They comprise certain histological types (adenoid cystic carcinoma [AdCC], secretory carcinoma, acinic cell carcinoma, tall cell carcinoma with reversed polarity, mucoepidermoid carcinoma, low-grade adenosquamous carcinoma, fibromatosis-like metaplastic carcinoma), some apocrine carcinomas, and TNBC that are rich in tumor-infiltrating lymphocytes (TILs) (3). After excluding these special tumor types, all TNBC tumors larger than 0.5 cm are candidates for systemic chemotherapy treatment (4, 5). The numerous treatment options for breast cancer are directed at therapeutic targets such as HR and HER2, which are absent by definition in TNBC. Thus, until recently, the only systemic treatment option for TNBC was chemotherapy, which included anthracyclines, taxanes, and platinum-based regimens (6). Two decades after identifying intrinsic molecular subtypes of breast cancer (7, 8), new treatment strategies for TNBCs are emerging based on the molecular characteristics of the neoplasm and its microenvironment (9, 10). Today, immunotherapy, poly ADP ribose polymerase (PARP) inhibitors, and some antibody-drug conjugates (ADCs) are already part of the therapeutic arsenal for these tumors. In this scenario, classical and molecular pathology play important roles.

## 2 Molecular subtypes of triple-negative breast carcinomas

Intrinsic molecular subtype identification by gene expression data was a turning point for comprehension of the biology of breast cancer (7, 8). Although the molecular taxonomy recapitulated the immunohistochemical profile of most breast cancers, particularly estrogen-positive tumors, TNBC remained an exception. It lacks a therapeutic target and exhibits significant heterogeneity in terms of prognosis and chemotherapy response. Most TNBCs (about 80%) (11) fall into the basal-like (BL) molecular phenotype, although TNBC and basal-like tumors are not synonymous (12). Besides, there is an overlap in gene expression between the different intrinsic molecular subtypes (12), and some basal subgroups have been identified, such as epidermal growth factor receptor (EGFR) overexpressed, *BRCA1*-related tumors, and claudin-low (13). In their review, Diaz et al. discussed the interrelationships between TNBC, the basal molecular phenotype, and *BRCA1*-related tumors, justifying a tendency to consider these entities synonymous (12). The association between *BRCA*-mutations and the BL molecular phenotype has been well-known for two decades since the study conducted by Sorlie et al. (14). About 25% of TNBC cases carry BRCA1/2 mutations (15). Subsequently, not only germline/somatic *BRCA* mutations but also other homologous recombination defects (HRD) come to be better understood as essential characteristics in a subgroup of TNBC. This understanding applies to both the clinicopathological presentation and the use of biomarkers in therapeutic decision-making (16, 17).

EGFR is overexpressed in more than 50% of TNBC (18), and the immunostaining of its protein, along with basal cytokeratins 5/6, is considered a surrogate for basal intrinsic molecular (18, 19). However, EGFR-targeted therapy did not show any benefit in TNBC (20). One of the reasons for this resistance is the activation of human epidermal growth factor receptor 3 (HER3), which is one of the four members of the HER family, along with HER2, EGFR (HER1), and HER4, and the main dimerization partner of EGFR (21). Upregulation of HER3 or its ligand, neuregulin, could be demonstrated in cell lines of TNBC of the BL type but not in the claudin-low subtype (22). In this scenario, the development of a bi-specific antibody targeting EGFR and HER3 gains support (22, 23).

The molecular classification based on intrinsic molecular subtypes is effective in managing estrogen-positive and HER2-positive tumors. However, it is not sufficient for TNBC, which is a highly aggressive and heterogeneous group of tumors that lack therapeutic targets. Lehmann et al.’s studies provided significant advancements in the molecular classification of these tumors (9, 10). They initially analyzed and clustered gene expression profiles into six subgroups, and further, they selected representative cell lines from each subtype to serve as models for different targeted therapies (9). After the initial classification, they separately analyzed tumor cells, tumor-associated stromal cells and TILs. They identified four tumor molecular subtypes and two additional gene expression patterns derived from lymphocytes and stromal cells. The molecular tumor subtypes were basal-like 1 (BL1), basal-like 2 (BL2), mesenchymal-like (MES), and luminal androgen receptor (LAR). The immunomodulatory (IM) subtype expresses genes involved in immune cell (IC) processes that originate from the population of TILs (10). The main molecular features of these subtypes are summarized in **Table 1**. A subtype of MES was the mesenchymal stem-like (MSL), which included genes linked to growth factor signaling pathways, such as inositol phosphate metabolism, EGFR, PDGF, calcium signaling, G-protein coupled receptor, ERK1/2 signaling, and ABC transporter and adipocytokine signaling. *PIK3CA-*activating mutations are frequent in the LAR subtype, indicating sensitivity to PI3K/AKT/mTOR pathway inhibitors. Important to note that *PIK3CA* mutations were also widespread in the MES subtype (47%) (9). Burstein et al., using mRNA expression and DNA profiling, identified four molecular subtypes: LAR, MES, basal-like immune activated (BLIA), and basal-like immune suppressed (BLIS). The LAR subtype was the same in the two classifications, and MES corresponded to most of MSL and some claudin-low of MES of Lehmann’s, while the remaining was included in BLIS. BL1 and BL2 of Lehmann’s were split between BLIA and BLIS, further rearranged according to immune gene activation (24). The four subtypes identified by Burstein et al. were confirmed to be distinct molecular entities, with BLIS and BLIA corresponding to the worst and better prognosis. Besides, their study provided insights into possible therapeutic targets. Identifying these molecular subtypes has revealed the heterogeneity of TNBC, allowing its stratification based on clinicopathological presentation and biomarkers, which we will discuss in the following sections.

**Table 1:** Molecular subtypes of TNBC and main molecular characteristics according to Lehmann et al. (9, 10).

Molecular subtype	Main molecular alterations
**Basal-like 1 (BL1)**	**Cell cycle and DNA response genes**
	• Enriched in genes involved in cell cycle (AURKA, AURKB, CENPA, BUB1, TTK, CCNA2, PRC1, MYC, NRAS, PLK1, BIRC5) • Enriched in DNA damage response genes (CHEK1, FANCA, FANCG, RAD54BP, RAD51, NBN, EXO1, MSH2, MCM10, RAD21, MDC1) • ATR/BRCA pathway • High Ki-67 mRNA expression)
**Basal-like 2 (BL2)**	**Growth factor pathways**
	• Enriched in growth factor signaling pathways genes (EGF, NGF, MET, Wnt/β-catenin, IGF1R pathways) • Enriched in growth factor receptor genes (EGFR, MET, EPHA2) • glycolysis/gluconeogenesis • higher expression of TP63 and MME (basal/myoepithelial markers)
**Luminal androgen receptor (LAR)**	**Hormonally regulated pathways**
	• Enriched in hormonally regulated pathways driven by the androgen receptor (AR)(high levels of AR mRNA and coactivators (DHCR24, ALCAM, FASN, FKBP5, APOD, PIP, SPDEF, and CLDN8))
**Mesenchymal-like (MES)**	**Epithelial-mesenchymal-transition (EMT) and growth factors pathways**
	• Genes involved in epithelial-mesenchymal-transition (EMT) • IGF/mTOR pathway • Cell mobility (regulation of actins by RHO) • ECM interaction • Cell differentiation pathways (Wnt pathway, anaplastic lymphoma kinase [ALK] pathway, and TGF-β signaling)
**Immunomodulatory (IM)**	**Immune cell processes**
	• immune cell signaling (TH1/TH2, NK cell, BCR signaling, DC, T-cell receptor signaling pathway) • cytokine signaling (IL12, IL7 pathways) • Immune signal transduction (NFKB, TNF, JAK/STAT) • ATR/BRCA pathway

## 3 Clinicopathological presentation of triple-negative carcinomas

Compared to non-TNBCs, TNBCs are more likely to occur in younger patients, have a higher grade, a larger tumor size, a higher clinical stage at diagnosis, and a poorer prognosis (25). Most are NST, but other histologies can be seen and present significant differences according to clinicopathological characteristics (26). In a retrospective cohort study using data from the Surveillance, Epidemiology and End Results (SEER) database, which included 19,828 patients, NST corresponded to 91.5% of cases, metaplastic to 3,2%, medullary to 1.5%, mixed NST-lobular to 1,2%, lobular to 1.0%, apocrine to 1.0% and adenoid cystic to 0.6%. Patients with lobular, metaplastic, and apocrine carcinomas tended to be older than those with NST carcinomas (proportion of 50 years or older, respectively, 89.6%, 79.4%, 89.7%, and 69.5%) (26).

AdCC and other rarest salivary gland-like tumors correspond to low-risk TNBC, with no/minimal metastatic potential (3). AdCC is generally unifocal and affects older women (2). It most frequently occurs as a classic variant, characterized by a low-intermediate nuclear grade and a mixture of tubular, cribriform, and solid patterns. The other variants are solid-basaloid and AdCC with high-grade transformation, both more aggressive than the classic variant. Breast AdCCs present the *MYB-NFIB* fusion gene, *MYBL1* rearrangements, and *MYB* amplification (27). They are the rarest and have a better prognosis than AdCCs in other localizations, such as the salivary gland and lacrimal gland, due to other molecular features (28).

Metaplastic carcinomas are morphologically very heterogeneous. They can present one or more metaplastic components, for example, squamous, spindle cells, mesenchymal, and matrix-producing cells (2). Most of the carcinomas in the group are high-grade, but there are also two subtypes, namely low-grade adenosquamous and fibromatosis-like metaplastic carcinomas, which are associated with an indolent course (3). Compared to non-metaplastic carcinoma, they are associated with worse outcomes, larger tumors, and less axillary involvement (29), besides being more resistant to chemotherapy (30, 31). Metaplastic carcinomas are classified as basal-like or claudin-low among the intrinsic molecular subtypes and MES subtypes, according to Lehmann et al. (9, 31). In a meta-analysis comparing metaplastic carcinoma with other TNBC, the authors observed that metaplastic carcinomas had a worse prognosis, higher age, larger tumors, and negative lymph nodes at the initial diagnosis. However, there were no statistically significant differences in the occurrence of distant metastasis, higher TNM stages, or higher histological grade (32). Just as their morphology is heterogeneous, molecular alterations are equally varied. The most often mutated genes are *TP53* and *PIK3CA*, while *MYC* and *EGFR* are the most commonly amplified, and the most common loss occurs in the *CDKN2A/CDKN2B* locus (30, 31).

A peculiar histological characteristic of some TNBCs is the medullary pattern, previously denominated medullary carcinoma, atypical medullary carcinoma, or carcinoma with medullary features. This pattern presents a well-circumscribed contour, pushing border, high histological grade, geographic-type necrosis, and prominent stromal TILs (2). Tumors with these characteristics belong to basal-like carcinomas with immune-activated molecular subtypes (IM, BLIA) (9, 24, 33, 34). Although the medullary pattern is classically associated with *BRCA1* mutations (2), it is possible that not only germline mutations but also sporadic BRCA mutations or mutations in other genes involved in DNA repair defects can contribute to immune activation and, consequently, the IM phenotype (35).

Both TNBC and HER2-positive carcinomas can exhibit apocrine differentiation, which is generally characterized by being estrogen receptor-negative and androgen-receptor (AR)-positive (2). Apocrine TNBCs, compared to other TNBC subtypes, are correlated with older age and present as smaller tumors with a lower grade, leading to a better prognosis (36). They correspond to the LAR subtype and 70% of TNBC with low Ki-67 proliferation (37).

Invasive lobular carcinomas (ILC) are rarely triple-negative. Analysis of 171,881 patients from the SEER database revealed that among 144,651 cases of NST, 16,433 cases of ILC, and 10,797 cases of mixed ductal and lobular, only 1.1% of ILC cases were classified as TNBC compared to 12.5% of ductal cases (38). Besides, the characteristics of triple-negative (TN) lobular and ductal breast cancer are different. Patients with ILC are older (71.7 y vs. 59.2 y), and tumors are more often AR-positive (94.7% vs. 50%), LAR subtype (39.3% vs. 17.1%), and have a lower Ki-67 index (17% vs. 39%) (39). Concerning molecular subtypes, triple-negatives ILC are more often luminal A (42.9% vs. 5.7%) and HER2 (39.3% vs. 17.1%) and less often basal-like (10.7% vs. 62.9%) (39). Besides the increased AR signaling, they frequently present alterations in the PI3K network, *ERBB2*, and *estrogen-related receptor alpha (ESRRA)* gene encoding, possibly implicated in significant predictive differences, particularly from ILC estrogen receptor-positive. Of note, the prognosis did not differ between ductal and triple-negative lobular carcinoma but was worse than non-triple-negative ILC (39).

## 4 Immunohistochemical profile of the molecular subtypes

After identifying the intrinsic molecular subtypes (7, 8), the surrogate immunohistochemical panel to define the BL tumors became the triple-negative phenotype (negative for estrogen receptor, progesterone receptor, and HER2), along with cytokeratin 5/6 and/or EGFR-positivity (40). Kumar et al. defined the BL1 subtype as positive for CK5/6 and/or CK4/14, and EGFR-negative, while BL2 was defined as EGFR-positive, irrespective of basal cytokeratins (41). In addition to basal cytokeratins and EGFR, other markers associated with the BL phenotype include p-cadherin, c-Kit, p63, and p16 (42, 43). However, as we could see from the molecular studies of Lehmann et al. (9, 10) and Burstein et al. (24), the heterogeneity of TNBC requires a more complex stratification. In the study conducted by Lehmann et al., it was found that 77% of TNBC cases were classified as basal, 12% as HER2-enriched, 4% as normal-like, 4% as luminal A, and 19.3% as luminal B. Among non-basal tumors, most of the LAR subtypes and some of the MES subtypes are included (10).

The LAR subtype represents 11 - 50% of TNBC. Compared to the other subtypes, it is more common in older women and expresses higher levels of AR. Additionally, it has a lower percentage of Ki-67 and frequently exhibits apocrine differentiation (10, 41, 44–46). Although HER2-negative, almost 50% of LAR tumors belong to the intrinsic molecular type HER2-enriched (45). The LAR subtype behaves similarly to other luminal tumors, with lower rates of pathologic complete response (pCR) and higher rates of axillary and bone metastases (47, 48). Immunohistochemistry (IHC) for AR is more than 10-fold higher in LAR subtypes, making it a useful tool for classifying TNBC (9). Generally, the cut-off to consider a TNBC of the LAR subtype is 10% or more (45, 49), although some authors consider at least 1% (41, 44). A more realistic characterization was proposed by Yoo et al., who found the best correlation between IHC and molecular subtype when AR scored Allred 8 (43).

Lehmann et al. demonstrated that the immune-activated phenotype is characterized by a high fraction of TILs (10). Harano et al. studied the frequency of the immune signature in the different molecular subtypes of TNBC, and they found 48%, 30%, 18%, and 0% among BL1, BL2, LAR, and MES, respectively (50). Besides its association with TILs, the IM subtype has been associated with the expression of programmed cell death 1 ligand (PD-L1) and CD8 (43, 45, 51). Yoo et al. defined the IM subtype by TILs above 70% (43), while Zhao et al. defined it as AR-/CD8+ (45). Kim et al. defined BL tumors by cytokeratin 5/6 and/or EGFR. These tumors were further stratified into BLIA and BLIS subtypes based on the expression of indoleamine 2,3-dioxygenase 1 (IDO1) and forkhead box C1 (FOXC1) (44). IDO1 is a cytosolic enzyme that degrades tryptophan and generates catabolites with important immune-regulatory functions (52). FOXC1 is essential for mediating various cancer stem cell traits, including cell proliferation, cell plasticity, partial epithelial-mesenchymal transition (EMT), cell migration, cell invasion, chemoresistance, and radioresistance (53). Upregulation of FOXC1 is higher in TNBC than in non-TNBC; it correlates with IHC expression and is a powerful negative prognostic factor (54). Kim et al. defined the BLIA phenotype as positive-IDO1/negative-FOXC1 and the BLIS phenotype as negative-IDO1/positive FOXC1 (44). The expression of FOXC1 was the adopted criterion for BLIS classification in different series (45, 49, 51). Lian et al. defined BLIS as having low levels of TILs, being AR-negative, CD8-negative, and FOXC1-positive (51). Besides its prognostic value, FOXC1 is suggested as a specific marker for TNBC. It is associated with basal markers and is inversely related to apocrine markers, such as Gross Cystic Disease Fluid Protein 15 (GCDFP-15) and AR (55).

Tumors of the MES subtype are AR-negative, CD8-negative and present frequent metaplastic features. They also show decreased expression of e-cadherin, claudin 3 and 7, and positive vimentin expression (41, 51). Doublecortin-like kinase 1 (DCLK1) is a novel biomarker of cancer stem cells and regulates tumorigenesis and EMT (56). The expression of this marker was used to define the MES subtype in the study conducted by Zhao et al. (45) and further explored by Leeha et al. (49). Lian et al. characterized the MES subtype as AR-negative, CD8-negative, and FOXC1-negative (51) expressions, while Yoo et al. defined tumors as AR-negative with less than 20% of TILs (43). The immunohistochemical profile, as summarized by different authors, is presented in **Table 2**.

**Table 2:** Immunohistochemical markers of the molecular subtypes of triple-negative breast carcinomas.

Molecular subtypes	Immunohistochemical profile
Basal-like	• Positive CK5/6 and/or CK14, and/or EGFR (40) • Positive p-cadherin, c-Kit, p63 (42) • AR-negative and p16 diffuse/strong (43)
Basal-like 1 (BL1)	• Positive CK5/6 and/or CK14, and negative EGFR (41)
Basal-like 2 (BL2)	• Positive EGFR, irrespective of CK5/6 or CK14 (41)
Basal-like immune activated (BLIA)	• CD8 (43, 45, 49, 51) • PD-L1 (43, 51) • Positive-IDO1/negative-FOXC1 (44)
Basal-like immune suppressed (BLIS)	• Negative-IDO1/positive-FOXC1 (44)
Mesenchymal (MES)	• Decreased expression of e-cadherin/claudin 3 and 7, and expression of vimentin (41) • Positive-DCLK1 (45, 49) • AR-negative/CD8-negative/FOXC1-negative (51)
Luminal androgen receptor (LAR)	• AR-positive (9, 41, 43–46)

CK, cytokeratin; EGFR: Epidermal Growth Factor Receptor; AR, androgen receptor; PD-L1, programmed cell death 1 ligand 1; IDO1, indoleamine 2,3-dioxygenase 1; FOXC1, forkhead box C1; DCLK1, Doublecortin-like kinase 1.

As we can remark, there has yet to be a consensus on surrogate markers for molecular classification, except for the LAR and BLIA subtypes. The AR expression plays a more precise role in defining the LAR subtype, even when using different cut-offs. Additionally, TILs and other immune molecules, such as CD8, define the IM phenotype.

## 5 Current therapeutic options

The molecular heterogeneity of TNBC, is a major challenge in the therapeutic management of these tumors. This has led to the initiation of numerous clinical trials, some of which have already been incorporated into clinical practice. **Table 3** summarizes the current systemic therapeutic options based on Phase III clinical trials.

**Table 3:** Current therapeutic options for triple-negative breast cancers based on Phase III clinical trials.

Trial	Number of patients	population	Agent
**CREATE-X** **NCT00130533**	286*	Post neoadjuvant therapy in patients with residual disease	Capecitabine
**OlympiAD** **NCT02000622**	302	Germline BRCA 1/2 mutation, metastatic disease, <= 2 prior chemotherapies	Olaparib
**EMBRACA** **NCT01945775**	130*	Germline BRCA 1/2 mutation, locally advanced/metastatic disease, <= 3 prior chemotherapies	Talazoparib
**OlympiA** **NCT02032823**	1,509*	Germline BRCA 1/2 mutation, high-risk disease (non-pCR, positive lymph nodes, >2 cm)	Olaparib
**Impassion130** **NCT02425891**	902	Untreated locally advanced/metastatic**	Atezolizumab+nabpaclitaxel
**KEYNOTE-355** **NCT02819518**	1,372	Untreated locally advanced/metastatic disease***	Pembrolizumab+chemotherapy
**KEYNOTE-522** **NCT03036488**	1,174	Untreated early high-risk disease	Pembrolizumab+chemotherapy
**ASCENT** **NCT02574455**	529	Locally advanced/metastatic disease, relapsed after at least two prior chemotherapies	Sacituzumab govitecan
**Destiny-Breast04** **NCT03734029**	63*	unresectable or metastatic HER2-low breast cancer, with 1-2 prior chemotherapies	Trastuzumab deruxtecan

*population of TNBC in the study; **Benefit for tumors with PD-L1, clone SP142, expressed in 1% or more immune cells; ***Benefit for tumors with PD-L1, clone 22C3, with CPS (combined Positive Score) of 10 or more.

### 5.1 Standard treatment

TNBCs measuring 0.5 cm or larger and/or those with positive lymph nodes are candidates for chemotherapy due to their inherent high risk. The standard treatment for patients with high-risk tumors involves incorporating taxanes (paclitaxel or docetaxel) into anthracycline-based regimens (cyclophosphamide plus doxorubicin) (5). For patients with stages II and III, neoadjuvant chemotherapy is preferred to potentially reduce locoregional surgical extension and tailor subsequent therapy based on pathologic response. This is because residual disease is associated with a higher risk of relapse. At this point, it is important to emphasize the significance of the pathologic evaluation of surgical specimens following neoadjuvant therapy. Pathologic assessment of response is crucial in defining pCR and quantifying residual disease, which in turn impactsprognosis and therapeutic decisions (57). The Capecitabine for Residual Cancer as Adjuvant Therapy (CREATE-X) trial has shown that for patients with TNBC who underwent standard neoadjuvant therapy with anthracycline, taxane, or both, and had residual invasive disease after treatment, the addition of capecitabine was associated with a better prognosis. Compared to the control group, the rate of disease-free survival was 69.8% in the capecitabine group versus 56.1% (hazard ratio for recurrence, second cancer, or death, 0.58; 95% confidence interval (CI), 0.39 - 0.87), and the overall survival rate was 78.8% versus 70.3% (hazard ratio for death, 0.52; 95% CI, 0.30 - 0.90) (58).

### 5.2 Poly ADP ribose polymerase (PARP) inhibitors

In 2018, the treatment of TNBC began to undergo significant changes. On January 12, 2018, the Food and Drug Administration (FDA) approved olaparib, a PARP inhibitor () for patients with HER2-negative metastatic breast cancer who have germline BRCA-mutations (gBRCAm) and have been treated with chemotherapy in the neoadjuvant, adjuvant, or metastatic setting (59). The decision was based on the clinical trial titled “Olaparib Monotherapy Versus Physician’s Choice Chemotherapy in the Treatment of Metastatic Breast Cancer Patients With Germline BRCA1/2 Mutations (OlympiAD)” (NCT02000622). This Phase III study involved 302 patients who were randomly assigned to receive either olaparib or a physician’s choice chemotherapy treatment. Olaparib monotherapy provided a significant benefit over standard therapy, with a median progression-free survival that was 2.8 months longer and a risk of disease progression or death that was 42% lower than standard therapy (60). On October 16, 2018, the FDA approved talazoparib, another PARP inhibitor, for the treatment of gBRCAm HER2-negative locally advanced or metastatic breast cancer (61). This approval was based on the findings of the EMBRACA study (NCT01945775) (62). On March 11, 2022, the FDA approved olaparib for the adjuvant treatment of patients with BRCA mutations presenting HER2-negative high-risk early breast cancer who completed definitive local treatment and neoadjuvant or adjuvant chemotherapy. The decision was based on the clinical trial OlympiA (NCT02032823), a Phase III study involving 1,836 patients with gBRCAm who were randomly assigned to receive either olaparib or a placebo. Olaparib was associated with longer survival free of invasive or distant disease than the placebo (63). Based on the rationale for PARP- inhibitors’ action in gBRCAm patients, other similar drugs, such as rucaparib, veliparib, and niraparib, have also been studied in clinical trials (64).

### 5.3 Immunotherapy

TNBC entered the immunotherapy era in 2019 after the initial results of the clinical trial IMpassion130 (NCT02425891), a multicenter, international, double-blinded, randomized study for unresectable locally advanced or metastatic TNBC. In this study, 902 untreated patients were randomized (1:1) to receive the immune checkpoint inhibitor (ICI) atezolizumab, an engineered humanized IgG1 monoclonal antibody anti-PD-L1, in combination with albumin-bounded paclitaxel (nab-paclitaxel) or placebo until disease regression or unacceptable toxicity. The median progression-free survival was 7.2 months with atezolizumab plus nab-paclitaxel, which was higher than placebo plus nab-paclitaxel (5.5 months) (hazard ratio for progression or death 0.80; 95% CI, 0.69-0.92; p=0.002). A clinical benefit was particularly evident with atezolizumab – nab-paclitaxel in the subgroup of patients with PD-L1–positive tumors. These patients had a median progression-free survival of 7.5 months with atezolizumab – nab-paclitaxel vs. 5.0 months with placebo – nab-paclitaxel (hazard ratio for progression or death, 0.62; 95% CI, 0.45 - 0.86). Additionally, they had a median overall survival of 25.0 months vs. 15.5 months (hazard ratio for death, 0.62 [not statistically tested]) (65). After these results, on March 8, 2019, the FDA granted accelerated approval to atezolizumab in combination with nab-paclitaxel for patients with unresectable locally advanced or metastatic TNBC whose tumors express PD-L1 tumor-infiltrating ICs covering ≥ 1% of the tumor area, as determined by the Ventana PD-L1 (SP142) assay (66). However, in September 2021, the pharmaceutical company announced that it had voluntarily decided to withdraw the U.S. accelerated approval for atezolizumab in combination with nab-paclitaxel for the treatment of unresectable locally advanced or metastatic TNBC (67). This decision did not affect the indications for atezolizumab for other tumors, including TNBC outside the United States.

Pembrolizumab, another ICI, is a humanized IgG4 anti-PD-1 drug that has been approved for breast cancer treatment. Two significant studies prompted the FDA’s approval of pembrolizumab in combination with chemotherapy for TNBC on July 26, 2021 (68). The clinical trial KEYNOTE-355 (NCT02819518), was a randomized (2:1) Phase III study that compared pembrolizumab plus chemotherapy to placebo plus chemotherapy for patients with untreated locally recurrent inoperable or metastatic TNBC. The trial involved 1372 patients. Among patients with tumors with a PD-L1 (22C3) combined positive score (CPS) of 10 or more, the median progression-free survival was 9.7 months with pembrolizumab-chemotherapy and 5.6 months with placebo-chemotherapy (hazard ratio for progression or death, 0.65; 95% CI, 0.49–0.86; p=0.0012) (69). The overall survival was evaluated in 847 patients with a median follow-up of 44.1 months. The addition of pembrolizumab in patients with tumors PD-L1 (22C3) CPS 10 or higher resulted in a longer overall survival (23.0 months vs. 16.1 months, hazard ratio for death, 0.73; 95% CI, 0.55 - 0.95; p= 0.0185) (70). The clinical trial KEYNOTE-522 (NCT03036488) investigated patients with early untreated high-risk TNBC (tumor size >1 cm up to 2 cm with positive lymph node or tumor size > 2cm regardless of node status). The patients were randomized (2:1) to receive pembrolizumab plus chemotherapy or placebo plus chemotherapy irrespective of tumor PD-L1 expression. Among the first 602 patients, the pCR rate was 64.8% (95% CI, 59.9-69.5) in the pembrolizumab-chemotherapy group and 51.2% (95% CI, 44.1-58.3) in the placebo-chemotherapy group (p<0.001) (71). In the interim analysis of 1174 patients, the event-free survival at 36 months was 84.5% (95% CI, 81.7- 86.9) in the pembrolizumab–chemotherapy group and 76.8% (95% CI, 72.2-80.7) in the placebo–chemotherapy group (hazard ratio for event or death, 0.63; 95% CI, 0.48-0.82; p<0.001) (72).

These trials using ICIs were fundamental to consolidating immunotherapy in TNBC. However, we have to admit that the role of the PD-L1 as a biomarker is intriguing. First, the biomarker did not matter in early-stage tumors. A possible explanation is that the mechanisms of immune inhibition, including PD-1/PD-L1, are not fully developed in the early stages of the disease. Second, although both trials with advanced disease are very similar, they use different PD-L1 tests that are not equivalent. Finally, considering the high complexity of the immune mechanisms, how can only one biomarker select cases for immunotherapy?

### 5.4 The era of antibody-drug conjugates (ADCs)

ADCs are a rapidly expanding group of new therapies that aim to deliver cytotoxic drugs using molecules selectively expressed in tumors. ADCs are composed of a monoclonal antibody that binds directly to an antigen in tumor cells. The payload typically consists of a cytotoxic agent, and there is a linker between the antibody and the payload. This engineering allows for the effective delivery of the cytotoxic agent directly into the tumor with fewer side effects. The basis of this mechanism motivated the designation of a “biological missile” for this group of drugs (73). The first ADC approved for breast cancer, and also the first for solid tumors, was ado-trastuzumab emtansine (TDM1). This drug is composed of an anti-HER2 monoclonal antibody linked with mertansine (DM1) via a succinimidyl-4-(N-maleimidomethyl)cyclohexane-1-carboxylate (SMCC) linker (73, 74). TDM1 is now used in HER2-positive tumors as both second-line and first-line treatment options, providing significant benefits, especially for patients with brain metastases (75).

The efficacy of ADCs depends on the attributes of their three components: the antibody, payload, and linker. Soluble linkers, for example, permit the payload to be released and cross the membrane acting in neighboring cells, even those that do not present the target antigen (73). Novel ADCs such as bispecific antibodies, dual-payload, and smaller molecules are being developed and tested to improve their actions. Bispecific antibodies can target either different sites of the same antigen or two different antigens. Dual-payload ADCs use two different cytotoxic and synergic drugs, minimizing drug resistance. A problem with the ADCs is their high molecular weight, which makes it difficult for them to penetrate tumors. As a result, only a small fraction of ADCs are able to reach tumor cells. A strategy to minimize this problem is to conjugate the payload with a fragment of the whole antibody, which reduces the molecular weight of ADCs and improves their penetration and drug delivery (73). Currently, two ADCs are used in TNBC (sacituzumab govitecan-hziy and fam-trastuzumab deruxtecan-nxki), and others are under investigation.

#### 5.4.1 Sacituzumab govitecan

This ADC is composed of sacituzumab, a humanized anti-trophoblast cell-surface antigen 2 (TROP2) monoclonal IgG1 kappa antibody, coupled with govitecan, a topoisomerase I inhibitor irinotecan, using the hydrolyzable CL2A linker (76).

TROP2 is a transmembrane glycoprotein initially characterized as a cell surface marker of trophoblast cells. It has since been implicated in cellular self-renewal, proliferation, invasion, and survival (76). It belongs to the epithelial cell adhesion molecule (EpCAM) family and is encoded by the tumor-associated calcium signal transducer 2 (*TACSTD2*) gene on chromosome 1p32. It is expressed at high levels by trophoblast cells, normal multistratified epithelia, and various cancer types, including TNBC (77).

Sacituzumab govitecan was approved by the FDA in April 2021 for metastatic TNBC that had received at least two prior therapies, based on the results of Phase III clinical trial ASCENT (NCT02574455) (78). In this trial, 529 patients with unresectable locally advanced or metastatic disease, who had relapsed after receiving at least two prior chemotherapies, were randomized (1:1) to either receive sacituzumab govitecan or physician’s choice of single-agent chemotherapy. The median progression-free survival for patients receiving sacituzumab govitecan was 4.8 months (95% CI 4.1-5.8), compared with 1.7 months (95% CI, 1.5-2.5) in those receiving chemotherapy (hazard ratio 0.43; 95% CI, 0.35-0.54; p<0.0001). The median overall survival was 11.8 months (95% CI, 10.5-13.8) and 6.9 months (95% CI, 5.9-7.6), respectively (hazard ratio 0.51; 95% CI, 0.41-0.62; p<0.0001) (79). Among these patients, 61 were included with brain metastasis and 54 with hormonal receptor-positive/HER2-negative tumors at initial diagnosis (79, 80). The results were similar in the subgroup without an initial TN phenotype (80).

The clinicians welcomed the ASCENT study because it was explicitly designed for TNBC. Besides, the trial included cases with a TN phenotype only in the recurrence, a not uncommon condition.

#### 5.4.2 Trastuzumab deruxtecan

Trastuzumab deruxtecan is a HER2-target ADC comprised of a humanized anti-HER2 monoclonal antibody, a tetrapeptide-based cleavable drug linker, and a topoisomerase I inhibitor payload. This ADC is characterized by a potent payload with a high drug-antibody ratio (8:1), a tumor-selective cleavable linker, a stable linker-payload, a short half-life payload, and a bystander effect (81). These features are associated with efficacy in tumors with low levels of HER2 and tumors with a heterogeneous distribution of HER2-positive cells (81). Trastuzumab deruxtecan was approved in early 2020 for the treatment of pretreated HER2-positive metastatic tumors (82). On August 5, 2022, the FDA approved trastuzumab deruxtecan for patients with unresectable or metastatic HER2-low breast cancer. This type of cancer is defined by IHC as 1+ or 2+/non-amplified. The approval is for patients who have received prior chemotherapy in the metastatic setting or developed disease recurrence during or within six months of completing adjuvant chemotherapy (83). Although most HER2-low breast cancers are HR-positive, the study also included TNBCs (84). The FDA approval for HER2-low breast cancer was based on the Destiny-Breast04 clinical trial (NCT03734029). This multicenter clinical trial included 557 patients with unresectable or metastatic HER2-low breast cancer. The patients were randomized (2:1) to receive either trastuzumab deruxtecan or the physician’s chemotherapy choice. The study included 494 HR-positive tumors and 63 TNBC (85). The median progression-free survival in the overall population was 9.9 months (95% CI, 9.0-11.3) in the trastuzumab deruxtecan group and 5.1 months (95% CI, 4.2-6.8) for those receiving chemotherapy (hazard ratio 0.50; 95% CI, 0.40-0.63; p<0.0001). The median overall survival (OS) was 23.4 months (95% CI, 20.0-24.8) in the trastuzumab deruxtecan arm versus 16.8 months (95% CI, 14.5-20.0) in the chemotherapy arm (hazard ratio 0.64; 95% CI, 0.49-0.84; p=0.001). Although the cohort of HR-negative individuals was small, its proportion was representative of the prevalence of this disease within the HER2-low population (85).

#### 5.4.3 Other ADCs under investigation

Upregulation of HER3, an important partner of EGFR, HER2, and HER4, is described in various cancers. Although the significance of HER3 is highlighted in HR-positive and HER2-positive breast cancers, TNBCs are also influenced by some of its functions (21). The HER3/HER2 and HER3/EGFR axes can be triggered in TNBC by different factors, such as upregulation of neuregulin or EGFR (21, 22, 86). It is associated with a poor prognosis and therapeutic resistance (21, 87, 88). However, the blockage of HER3 did not show any clinical benefit. In this scenario, ongoing trials investigate a promising strategy using the ADC patritumab deruxtecan (87, 88). Patritumab deruxtecan comprises a recombinant fully human antibody linked to a topoisomerase I inhibitor. The main ongoing clinical trials to evaluate this drug are NCT04610528 [A Window-of-opportunity Study of U3-1402, a HER3-targeting Antibody-drug Conjugate in Operable Breast Cancer According to ERBB3 Expression (TOT-HER3)]; NCT02980341 (Phase I/II Study of U3-1402 in Subjects With Human Epidermal Growth Factor Receptor 3 (HER3) Positive Metastatic Breast Cancer); NCT04965766 [Patritumab Deruxtecan (U3-1402) in Unresectable Locally Advanced or Metastatic Breast Cancer (ICARUS-BREAST)]; and NCT04699630 (A Study of U3-1402 in Subjects With Metastatic Breast Cancer).

The folate receptor alpha (FRα) protein is a member of the FR family, and it is located on cell membranes. FRα binds to folic acid and its derivatives, which becomes crucial during fetal development. After embryogenesis, it has limited expression in normal tissues (89). As folate plays a significant role in DNA replication and cell division, it is common to find FRα expressed in some aggressive cancers, including 20-80% of TNBC (90, 91). In most publications, it was associated with a poor prognosis (92, 93), although some authors found better outcomes (91). The hypotheses for these unexpected results were increased sensitivity to chemotherapy and the release of FR antigens, which provoked an immune response (91). Low expression in normal tissues and overexpression in carcinomas constitute an excellent combination for ADCs. Mirvetuximab soravtansine is an ADC composed of a humanized monoclonal antibody anti-FRα linked to a microtubule inhibitor. On November 14, 2022, the medicament received FDA approval for ovarian cancer patients with FRα-positive, platinum-resistant epithelial ovarian, fallopian tube, or primary peritoneal cancer who have undergone one to three prior systemic treatments (94). For breast cancer, the positive FRα expression was determined by the proportion score (PS) method and defined as ≥25% of cells having ≥1+ membranous expression by IHC (90).

## 6 Biomarkers

### 6.1 Ki-67

The antigen Ki-67, identified by immunohistochemistry and encoded by MKI67, is a nuclear protein expressed in all cell cycle phases except G0. In a systematic review conducted by van den Ende et al., high expression of Ki-67 was found to be a potent predictor of therapy response, although different cut-offs have been utilized (48). Zhu et al. found that a cut-off of 30% for Ki-67 was the most effective in defining independent prognostic groups, especially in stage I patients (95). Srivastava et al. reported similar results using the same cut-off (37). These authors studied 70 patients with TNBC who had a Ki-67 index of 30% or less. They observed low-grade tumors and enrichment by special histologies, with mostly apocrine tumors (70%), AR-positive (80%), and HER2-low (81%) (37). A meta-analysis conducted by Wu et al. included 35 studies with 7,716 patients, which demonstrated an association between high Ki-67 expression and poor outcomes (96). These authors identified a cut-off of 40% associated with a higher risk of recurrence and death.

Although a cut-off for Ki-67, either predictive or prognostic, is not as well defined for TNBC as it is for hormonal receptor-positive carcinomas, it offers additional information to individualize the management of cases.

### 6.2 Tumor-infiltrating lymphocytes

The microenvironment of solid tumors includes various cellular types, like ICs, which result from the interaction between the tumor and the immune system. Tumor cells differ from their normal counterparts in that they express different antigens. These antigens can be expressed on the cell surface as peptides bound to major histocompatibility class I (MHC-I), or they can be released by dying cells or secretion products. Immune activation depends on the capture and processing of tumor neoantigens by dendritic cells/antigen-presenting cells. The dendritic cells migrate to the lymph nodes to present the neoantigens to naïve T cells, thereby priming and activating them against the cancer-specific antigens. Activated lymphocytes migrate to the tumor site, interacting with tumor cells and binding their T-cell receptor to the antigen bound to MHC-I, ultimately killing them (97, 98). However, many factors can interfere with the efficacy of the immune response. Tumor antigens cannot be recognized as foreign, or they cannot be released. Depending on the type of antigen on the tumor surface, tumors can behave as themselves and induce a T regulatory response instead of an effector (97). Besides, during the immune cycle, the concurrent co-activation of both stimulatory and inhibitory immune checkpoints modulates the type of response (98, 99).

The number and composition of ICs in tumors are determined by immune activation and the mechanisms of immune suppression. Their presence indicates that the immune system has been activated, and their composition includes either pro-tumorigenic or anti-tumorigenic cells (100). However, even with a subset of negative immune regulators, the quantity of TILs is associated with prognosis and is predictive of chemotherapy response (101). Lehmann et al. identified the TILs as the population responsible for the gene expression of the IM molecular subtype (10).

The evaluation of TILs is based on hematoxylin-eosin-stained slides following the recommendations of the International TILs Working Group 2014 (100). Stromal TILs are expressed as the percentage of tumor stroma occupied by lymphocytic infiltrates without direct contact with tumor cells (102). In a study involving 607 patients with TNBC who received neoadjuvant chemotherapy from six randomized trials conducted by the German Breast Cancer Group trials, it was found that a 10% increase in TILs was associated with improved disease-free and overall survival rates. TNBC achieved pCR in 80/260 (31%) with low TILs (0-10%), 117/373 (31%) with intermediate TILs (11-59%), and 136/273 (50%) with high TILs (60% or more) (p<0·0001 for each group) (101). Two meta-analyses have confirmed these findings (103, 104). Higher TIL values are also associated with improved disease-free and overall survival in tumors treated with adjuvant chemotherapy (105). Luen et al. have shown that TILs in residual tumors post-neoadjuvant treatment are associated with longer recurrence-free and overall survival, particularly in cases with residual cancer burden (RCB) class II (106).

The TIL population includes different types of IC with a predominance of cytotoxic lymphocytes (CD8+) and forkhead box P3 (FOXP3) regulatory T lymphocytes. These cells are respectively involved in antitumor immunity and immune escape. Interestingly, even with at least two antagonistic populations, TILs have undeniable prognostic value (100, 101, 107). In general, the CD8+ component identifies better tumors that are more likely to achieve a pCR and are associated with a better prognosis (48). It is important to mention that all immune biomarkers, whether activators or suppressors, are correlated (48, 107, 108).

TILs are key players in immune reactions, and they have been widely studied in immunoncology. As a result, the International TILs Working Group 2014 (100) and the WHO Classification of Breast Tumors (2) have provided recommendations based on these evaluations. For now, the quantification of TILs plays a prognostic role. However, this information is expected to aid in selecting candidates for immunotherapy.

### 6.3 PD-L1

Immune checkpoints regulate the immune cycle in non-neoplastic conditions to prevent hyperactivity of cytotoxic T cells. They also serve as evasion mechanisms in neoplastic conditions. They correspond to receptor-ligand pairs that act as inhibitory or stimulatory pathways in the immune cycle (109). Programmed cell death 1 (PD-1) receptor and its ligands, PD-L1, and PD-L2 constitute an important inhibitory pathway that mediates the immune response in both normal and neoplastic conditions. Immunotherapy in TNBC is based on ICIs targeting PD-1 or PD-L1, which facilitate the reactivation of cytotoxic T cells to kill tumor cells. Atezolizumab, pembrolizumab, durvalumab, avelumab, and nivolumab, are examples of monoclonal antibodies designed to bind to PD-L1 or PD-1, inhibiting the axis and enabling the reactivation of T cells (110). The expression of PDL1 is a biomarker for these drugs, particularly in metastatic/advanced disease (65, 70). In early-stage disease, although the expression of PD-L1 has prognostic value, the efficacy of the treatment is independent of this biomarker (72). This may be because the mechanisms of evasion tend to manifest at a later stage. Besides predicting the response to ICIs, PD-L1 also predicts pCR after neoadjuvant therapy treatment using regimens including anthracycline/taxane (48).

PD-L1 is a transmembrane molecule expressed on tumor cells and/or tumor-infiltrating ICs, including dendritic cells and macrophages (111). Various immunohistochemical assays can assess PD-L1, each using different primary antibodies that target different epitopes of the molecule, both intracellularly and extracellularly. The staining pattern for tumor cells is membranous, while ICs exhibit a granular and punctate pattern. Besides, PD-L1 assays have been developed as predictive biomarkers for particular ICIs as companion diagnostics, each using distinct immunohistochemical platforms and interpretation systems (112). The companion diagnostic for atezolizumab is the SP142 assay, which utilizes a monoclonal rabbit antibody designed for use on the VentanaMark Ultra instrument. The interpretation requires at least 50 viable tumor cells with associated stroma. It is based on the IC score, expressed as a percentage of the tumor area occupied by PD-L1-positive ICs in the intratumoral and contiguous peritumoral areas. PD-L1-positive tumors are defined by IC≥1% (65, 112). The assay used for pembrolizumab is the 22C3 pharmDx from Agilent Dako, and the interpretation is based on the combined positive score (CPS), with a cut-off of ≥10 and requiring a minimum of 100 viable tumor cells. CPS is defined as the ratio of all positive cells (including any stain in tumor and IC) to the total number of viable tumor cells in the assessed area, multiplied by 100 (70, 112).

The comparison of three PD-L1 assays (SP142, 22C3, and SP263) in tumor samples from 614 patients with metastatic/advanced TNBC in the Impassion130 clinical trial showed no inter-assay analytical equivalency. SP142 (IC≥1%) corresponded to 46.4% of tumors, while 22C3 (IC≥1%) corresponded to 73.1%, 22C3 (CPS>1) corresponded to 80.9%, and 22C3 (CPS≥10) corresponded to 52.9%. Moreover, the population identified by the different assays, even after the analytical harmonization (22C3 CPS≥10), differed. 22C3 CPS≥10 missed 22.4% of SP142 IC≥1% cases (113). PD-L1 is the only biomarker approved for ICIs in TNBC, but there are many controversies surrounded its use in clinical practice. PD-L1 exhibits dynamic expression, heterogeneous distribution within the tumor area, and questionable reproducibility, particularly when borderline values are considered (114–116).

The PD-L1 test should be optimized to be more user-friendly. It is challenging for pathologists to work with different immunohistochemical antibodies/platforms, interpretation methods, and scoring systems, especially when considering the specific type of drug and tumor. The association with other immune biomarkers, such as TILs and tumor mutational burden (TMB), along with a general antibody for the immunohistochemical reactions, could provide a solution.

### 6.4 Other biomarkers for immunotherapy

Besides TILs and PD-L1, other biomarkers related to ICIs have been explored (117–120). TMB corresponds to the number of somatic mutations per megabase (mut/Mb) of DNA (119). On June 16, 2020, the FDA approved pembrolizumab for the treatment of patients with unresectable/metastatic tumors with high TMB (≥10 mut/Mb) that have progressed following prior treatment without satisfactory alternative options (121). High TMB is present in up to 5% of primary breast cancer cases and is more prevalent in metastatic disease (122–124). It is associated with a better prognosis in metastatic TNBC treated with ICIs (125) and is a potential biomarker for immunotherapy. Nevertheless, prospective clinical trials and methodological standardization are needed (118, 120).

Lymphocyte activating gene-3 (LAG-3) is an inhibitory immune checkpoint expressed on activated T lymphocytes (126). A meta-analysis conducted by Hu et al. found a favorable prognostic role for LAG-3+ lymphocytes in TNBC (126). On the other hand, its expression in tumor cells has been suggested as a resistance factor to ICIs (127). The prognostic and predictive values of LAG-3 deserve further study, considering the specific cell type expressing the molecule and its association with other factors that can affect it, such as TILs and other immune checkpoints. Moreover, it is difficult to exclude TILs from any evaluation of immune activation during ICI therapy. After all, the rationale behind ICI is to allow cytotoxic lymphocytes to perform their work.

### 6.5 HER2-low

HER2-low corresponds to the subgroup of HER2-negative breast cancer that expresses some degree of protein in the membrane as characterized by IHC 1+ or 2+/non-amplified according to the American Society of Clinical Oncology/College of American Pathologists (ASCO/CAP) 2018 guidelines, updated in 2023 (128, 129). Traditional HER2-targeted agents have no clinical benefit for this condition, but novel ADCs are changing the treatment paradigm (130). The clinical trial Destiny-Breast04 with trastuzumab deruxtecan showed impressive results in patients with unresectable or metastatic HER2-low breast cancer (85). Other ADCs with anti-HER2 antibodies are currently being developed and tested, such as trastuzumab duocarmazine, and bispecific antibodies (130). Although most HER2-low tumors are HR-positive, about 20% are TNBC, which opens up new treatment opportunities for them (84). The reported differences between HER2-score 0 and HER2-low, such as a lower Ki67 index, less often high grade, and reduced pCR, are influenced by the hormonal status and unrelated to the HER2-low status. When only HER2-low TNBC is analyzed, the pCR does not differ between HER2-score 0 and score 1+ (131). The low expression of HER2 without amplification predicts the trastuzumab deruxtecan response but it does not indicate a new entity or subtype of tumors (130). The HER2-low condition is unstable, exhibiting changes throughout the course of the disease and after neoadjuvant therapy, along with significant spatial and temporal discordances (132–134). There is a minimal prognostic difference between HER2-0 and HER2-low (135). These characteristics indicate that HER2-low is not a tumor subtype but rather a predictive condition.

The ASCO/CAP has updated the recommendations for HER2 testing and affirmed the previous 2018 guidelines (129). The panelists considered it premature to change the current terminology for low expression. However, they recommended including a footnote regarding the eligibility for treatment. They reinforced the care in the distinction between HER2-0 and HER2-1+, in terms of interpretation and pre-analytical procedures. The latter is fundamental for identifying low expressions (129). This posture is understandable since we only have the results of the Destiny-Breast04 study. In this clinical trial, the HER2-0 group was not studied, so we do not know the lower limit of low expression that may benefit from treatment. The Daisy trial (NCT04132960), a Phase II study of trastuzumab deruxtecan for advanced breast cancer, has contributed to a better understanding of biomarkers (136). This study, although small, enrolled 186 patients, of whom 39 had TNBC. It demonstrated that the effectiveness depends on the HER2 expression level, which occurs, albeit at a smaller scale, even in tumors with a score less than 1+. Besides, other factors can be involved in the resistance, such as the mutation of *SLX4*, a gene that encodes a DNA repair protein and regulates an endonuclease involved in the endocytosis of the ADC (136). HER2-protein as a target for ADC is fascinating, and both the Destiny-Breast04 and Daisy trials have proven to have a clinical impact. These studies highlighted the need for re-defining the HER2-negative tumors based on protein expression levels, which could be potentially benefit from trastuzumabe deruxtecan. However, we still don’t know how low we can go in the definition of HER2 status to maintain clinical benefit from ADCs. We can assume that any visible staining in IHC can be sufficient to select the eligible cases, or perhaps we will need to define them according to different criteria, such as protein quantification.

For now, the search for this biomarker can be preferable for biopsies since samples are generally better preserved than surgical specimens (137). Because of the heterogeneity of expression, primary or metastatic results can be considered when making therapeutic decisions (129, 132).

### 6.6 TROP2

Ambrogi et al. studied the expression of TROP2 by IHC in 702 consecutive breast cancer patients’ tumors. They showed that membrane localization is associated with poorer cancer patient survival. In comparison, intracellular expression is associated with less frequent disease relapse and better survival, suggesting that the activation state of TROP2 is a critical determinant of tumor progression (138). Izci et al. analyzed TROP2 staining in 589 tumors and observed high expression (H-score 201-300) in 97 (16.5%) cases, medium expression (H-score 100-200) in 149 (25.3%) cases, and low expression (H-score <100) in 342 (58.2%) cases, of these, 151 (25.6%) cases showed no staining (77). Unlike Ambrogi et al., Izci et al. did not demonstrate an association between TROP2 expression and survival. However, they found a significant association with lymphovascular invasion and lymph node involvement. One of the factors that could potentially explain the differences in survival between these two studies is the type of antibody used in the IHC reactions. Another factor is the correlation of TROP2 with the androgen receptor and apocrine histology, as demonstrated by Izci et al. Both the presence of the androgen receptor and apocrine features suggest the LAR molecular subtype. It is possible that the population studied by Izci et al. was enriched with the LAR subtype, which is known to be a less aggressive TNBC subtype.

The benefit of sacituzumab govitecan demonstrated in the ASCENT study was independent of expression of TROP2. However, favorable outcomes were higher with Sacituzumab govitecan in patients with high and medium TROP2 expression when treated with Sacituzumab govitecan (139). Nonetheless, a few cases (59) had a low H-score. The proportion of tumors with high/medium TROP2 expression was higher in the ASCENT trial (advanced tumors) than in the Izci et al. study (early and advanced tumors), possibly because there might be differences between early and advanced settings.

## 7 Final remarks

Despite the advancements in molecular knowledge of breast tumors that do not express HR and HER2, the term “triple-negative breast cancer” is widely established in clinical practice and the medical literature. We cannot deny that the name “TNBC” itself is not incorrect, although it is certainly insufficient for prognostic evaluation and therapeutic management. Shortly after identifying intrinsic molecular subtypes, there was a tendency to consider the triple-negative condition, the basal phenotype, and the germline BRCA mutation-associated tumors almost synonymous (13). The first step in understanding TNBC was to separate non-basal from basal-like tumors. Although the latter percentage varies among TNBC cases depending on the characteristics of the studied population, it typically corresponds to 70-80% (11). Lehmann et al. and Burstein et al. promoted an important advance in the molecular analysis and understanding of these tumors. They demonstrated that even the basal tumors do not form a homogeneous group. Instead, they involve different intracellular pathways that result in various patterns of immune system activation (9, 10, 24). Despite some overlap in molecular characteristics, the BL1 group includes most tumors with cell cycle disorders and DNA repair defects, including here *BRCA* mutations. On the other hand, the activation of growth factor signaling pathways characterizes BL2 tumors. Depending on the cellular dysfunction, the basal phenotype defines different patterns of immune activation that result in immunoactivated (BLIA) or immunosuppressed (BLIS) tumors (24).

Up to this point, we already have two groups of tumors with well-designed therapies: TNBC-immunoactivated and TNBC-associated with BRCA mutation (62, 63, 65, 70). The identification of these groups in clinical practice can be done without difficulty. For immune-activated tumors, we utilize TILs and IHC for PD-L1, CD8, and IDO1, in addition to assessing TMB, immune signatures, and other relevant factors. In the case of BRCA-associated tumors,it is crucial to prioritize hereditary genetic testing, particularly for patients under the age of 50 years. These patients, even without a family history of breast or ovarian cancer, have a >10% chance of having germline *BRCA* mutation, and this frequency progressively increases as age decreases (140).

More recently, several ADCs have been identified for the treatment of TNBC. Some of these ADCs have already been approved and target HER2-low and TROP2. Others show great promise and target HER3 and the FRα. Additionally, several others ADCs are currently undergoing initial tests (79, 85, 87, 90).

**Figure 1:**
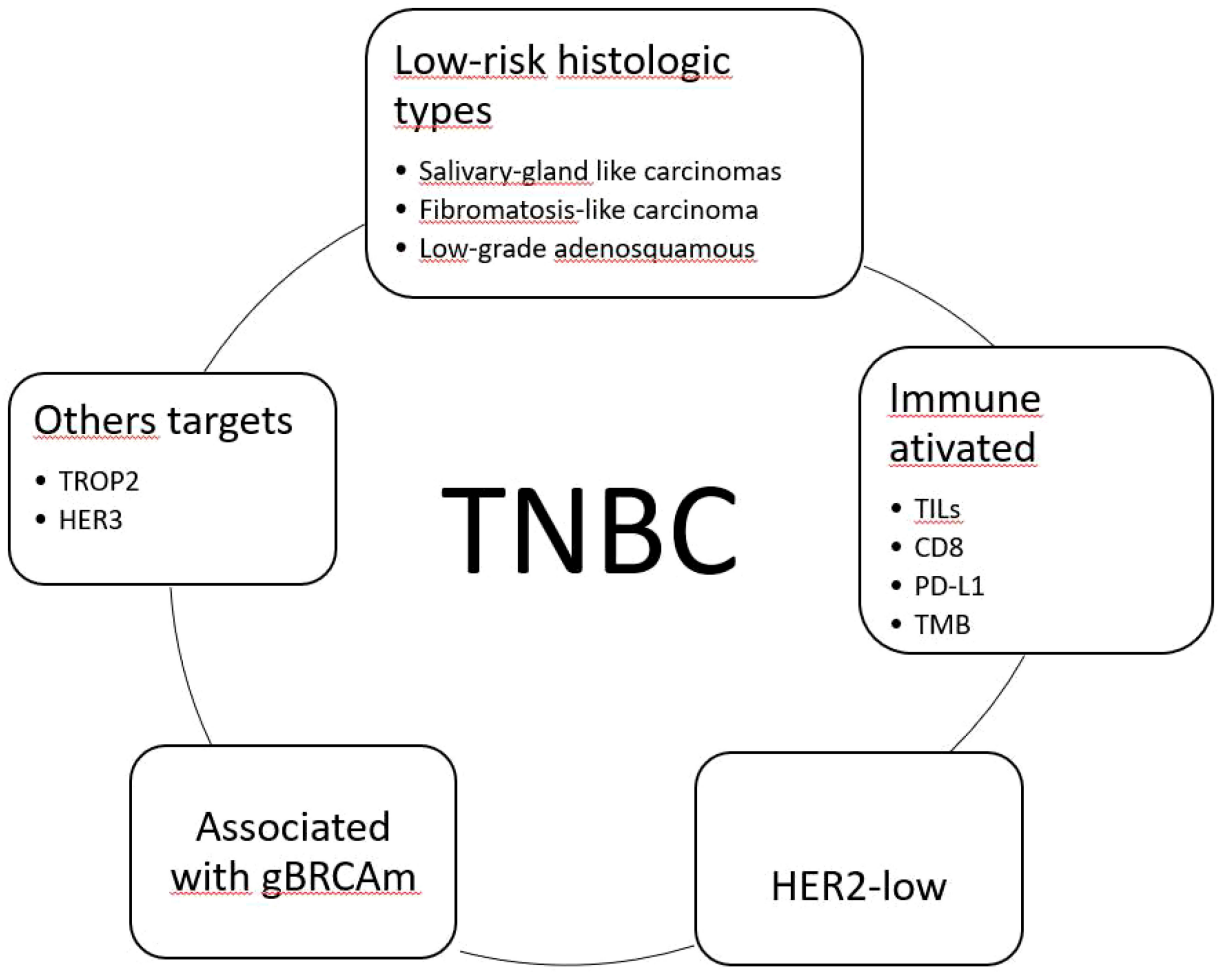
Classification of triple-negative breast carcinomas (TNBC) considering available therapeutic options. TILs, tumor-infiltrating lymphocytes; TMB, tumor mutational burden; HER2-low, HER2 immunohistochemical score 1+/2+ non-amplified; gBRCAm, germline BRCA1/2 mutation; TROP2, trophoblast cell-surface antigen 2; HER3, human epidermal growth factor 3.

The TNBC issue is extensive, with many aspects meriting discussion, including clinical, histological, molecular, and therapeutic aspects. This review was based on articles selected by the author, and it has some limitations, such as the lack of mechanisms involved in drug resistance, for example. One reason is that this review was based on the pathologist’s perspective, who is responsible for the initial diagnosis of breast cancer type and is concerned about which information is now essential and how to understand TNBC as a group of different diseases. **Figure 1** illustrates the TNBC subgroups according to therapeutic possibilities.

One cannot ignore the exciting trajectory of TNBC, initially defined by the lack of biomarkers and currently heading toward countless therapeutic possibilities that only depend on identifying the appropriate adjective based on its molecular characteristics. So, it is time to no longer refer to TNBC, but rather specify which TNBC we are discussing.

## Author contributions

FC is responsible for all steps of the article.
